# Proceedings of the American Pharmaceutical Association, at the Fifth Annual Meeting, Held in Baltimore, Sept. 1856

**Published:** 1857-02

**Authors:** 


*Proceedings of the American Pharmaceutical Association, at the Fifth Annual Meeting, held in Baltimore, September,* 1856; *with a List of Officers and Members.*

We have here a pamphlet of ninety pages, containing the proceedings of the Association; the reports of committees; various communications in relation to the active principles of many remedial agents, their mode of preparation and preservation; the subjects assigned to the investigation of committees for the ensuing year, &c. It is a highly-interesting and valuable pamphlet, especially to pharmaceutists and druggists.

